# Virulence of *Beauveria* sp. and *Metarhizium* sp. fungi towards fall armyworm (*Spodoptera frugiperda*)

**DOI:** 10.1007/s00203-023-03669-8

**Published:** 2023-09-07

**Authors:** Nonthakorn (Beatrice) Apirajkamol, Timothy Michael Hogarty, Bishwo Mainali, Phillip Warren Taylor, Thomas Kieran Walsh, Wee Tek Tay

**Affiliations:** 1 Applied BioSciences, Macquarie University, Sydney, NSW 2109 Australia; 2 Black Mountain Laboratories, Commonwealth Scientific and Industrial Research Organisation, Canberra, ACT 2601 Australia

**Keywords:** Entomopathogenic fungi, Agricultural pest, Biocontrol, Sustainable pest management, Bioassay

## Abstract

The development of effective pest management strategies for *Spodoptera frugiperda* is a high priority for crop protection across its invasive ranges. Here, we examined six *Beauveria* and five *Metarhizium* fungal isolates against this pest. Two *Beauveria* isolates (B-0571, B-1311) induced high mortality toward 3rd and 6th instar caterpillars and adults. For B-0571 mortality was 82.81 ± 5.75%, 61.46 ± 6.83%, and 93.75 ± 3.61%, and 73.72 ± 2.51%, 71.88 ± 5.41%, and 97.92 ± 2.08% for B-1311, with deaths in caterpillars largely occurring under 24 h (3rd instar control 0.74 ± 0.33%, B-0571 73.96 ± 7.85% and B-1311 62.08 ± 3.67%; 6th instar control 0%, B-0571 66.67% ± 11.02% and B-1311 62.5% ± 9.55%). Infection from both Beauveria isolates fully prevented reproduction in surviving *S. frugiperda* females. In contrast, all five *Metarhizium* isolates tested and the remaining four *Beauveria* isolates exhibited lower virulence. The discovery of two highly virulent *Beauveria* fungal isolates to *S. frugiperda* opens avenues to develop novel biological control tools against this highly invasive pest.

**Supplementary Information:**

The online version contains supplementary material available at 10.1007/s00203-023-03669-8.

## Introduction

The fall armyworm, *Spodoptera frugiperda* (J.E. Smith) (Lepidoptera: Noctuidae), is an invasive moth species that was detected in the Torres Strait Islands, Australia, in late January 2020 (Tay et al. 2023a). By the end of 2020, it had been detected widely across mainland Australia (International Plant Protection Convention 2020; Government of Western Australia 2021; Tay et al. 2022a). *S. frugiperda* is native to the tropical regions of the American continents (Southern United States to Argentina) where it is a serious pest of agricultural production (Capinera 1969; Andrews 1980). In 2016, *S. frugiperda* was confirmed outside of its native range in West Africa followed by reports of significant economic damage to maize crops. In 2017, 12 African countries lost approximately 8.5–21 M tonnes of maize (2.5–6.3B USD) to this species (Day et al. 2017). *S. frugiperda* has been recorded on over 350 plant species and commonly feeds on sorghum, wheat, cotton, sugarcane, and various vegetables (Montezano et al. 2018), however, in the invasive ranges’ maize has been the preferred host although increasingly impacting other economic crops. *S. frugiperda* has a high reproductive rate; a single female can lay more than 2000 eggs in her lifetime (Capinera 1969). It is also able to disperse over long distances, especially with prevailing winds, when hosts are limiting (Westbrook et al. 2016), and in response to season and weather (Westbrook et al. 2019). Due to economic damage, polyphagy, fecundity, and dispersion from mobility, and transportation of contaminated live plant product (Tay et al. 2022b, 2023b), *S. frugiperda* has quickly become a major threat to agricultural production across its invasive ranges (Goergen et al. 2016; Cock et al. 2017; Ganiger et al. 2018; du Plessis et al. 2018; Trisyono et al. 2019; Agboyi et al. 2020; Fan et al. 2020; Kandel and Poudel 2020; Lee et al. 2020; Piggott et al. 2021; Zaimi et al. 2021; Zhou et al. 2021; Tay et al. 2022a; Wu et al. 2022). There is now high demand for effective and sustainable management tools to reduce economic impact of this invasive pest.

Globally, management strategies for *S. frugiperda* have relied heavily on synthetic insecticides and genetically modified Bt crops, which contributed to the development of insecticide resistance (Togola et al. 2018). *S. frugiperda* populations showed resistance to several synthetic insecticides in both their native range (Yu et al. 2003; León-García et al. 2012; Garlet et al. 2021) and in recently colonised regions (e.g., indoxacarb, chlorpyrifos, and malathion, Zhao et al. 2020; Zhang et al. 2020; Kulye et al. 2021; to methomyl, Tay et al. 2022a). Additionally, resistance to Bt proteins including various Cry1 (Storer et al. 2010; Jakka et al. 2014; Huang et al. 2014; Gutierrez et al. 2015; Monnerat et al. 2015; Leite et al. 2016), Cry2Ab2 (Niu et al. 2016), and Vip3Ac (Yang et al. 2018) have also been detected in their native range. The rapid spread of *S. frugiperda* across the recent invasive range likely reflected multiple introduction events (Schlum et al. 2021; Tay et al. 2022a, b; Tay et al. 2023b). It is likely that the invasive *S. frugiperda* populations carry varying levels of susceptibility towards synthetic insecticides and Bt transgenic plants (Eriksson 2019; Worku Bogale and Andargie 2019; Boaventura et al. 2020; Zhao et al. 2020; Lv et al. 2021; Zhang et al. 2021; Kulye et al. 2021; Tay et al. 2022a), and to-date, no resistance to Bt proteins has been detected in invasive *S. frugiperda* populations (Botha et al. 2019; Liang et al. 2021; Tay et al. 2022b) although the situation may change especially if on-going introduction of novel populations from native ranges continues. Current management strategies would benefit from incorporation of alternative biological control options to improve overall efficacies that also concurrently reduces selection pressure for insecticide resistance.

Biological control, which utilizes a pest’s natural enemies including predators, parasites (e.g., parasitic wasps, parasitic nematodes), competitors and pathogens (e.g., fungi, bacteria, and viruses) to suppress populations (Flint et al. 2002), is a pillar of integrated pest management (IPM) strategies. Unlike most synthetic insecticides, biological control agents are often host specific and hence tend to have less effect on non-target species and the broader environment (Dodd 1959; Muthukumar et al. 2007; Geiger et al. 2010; Kumar and Singh 2015; Mills et al. 2016; Blossey et al. 2018; Cernava et al. 2019; Nawaz et al. 2021). There are many examples of successful biological control agents globally including parasitic wasps (*Trichogramma* spp.) to control lepidopteran pests in China (Liu et al. 2014; Wang et al. 2014), cactus moth (*Cactoblastis cactorum*) to control prickly pear in Australia (Dodd 1959), and entomopathogenic fungus (*Beauveria bassiana*) to control coffee berry borer (*Hypothenemus hampei*) in Colombia (Aristizábal et al. 2016) and many more (van Lenteren et al. 2018). In India and Uganda*,* various species of parasitic wasps and nematodes, predatory insects, and fungi have been implicated as playing important roles in suppressing *S. frugiperda* populations (Firake and Behere 2020; Visalakshi et al. 2020; Otim et al. 2021), highlighting the potential value of biological control, particularly within broader IPM systems.

With respect to efficiency, sustainability, and economic perspectives, entomopathogenic fungi (EPF) that can infect and utilize insects as a host for development as part of their life cycle (Shah and Pell 2003; Dillman et al. 2012), are well-suited as biological control tools. Under ideal conditions, EPF can cause epizootics to significantly reduce the population of insect pests by up to 90% (Lovett and St. Leger 2018). The majority of EPF infect insects by enzymatically degrading the insect cuticle follow by penetrating the hemocoel, where they often cause nutritional deficiency and host death (Shah and Pell 2003; Trakimas et al. 2019). In some cases, EPF also produce bioactive compounds that accelerate infection by suppressing the host’s immune system and/or being toxic to the host itself or help supressing microbial competitors (Donzelli and Krasnoff 2016; Mondal et al. 2016). They germinate from the insect carcass and disperse to reinfect other insect hosts. EPF can provide an excellent alternative to synthetic insecticides as both a standalone tool or as an IPM component (Roberts and Hajek 1992). In 2016, approximately 1604 species and isolates of EPF have been identified globally (St Leger and Wang 2010; Araújo and Hughes 2016), and over 170 EPF-based biopesticides commercially developed (both registered and unregistered) worldwide to manage diverse insect pests (e.g., beetles, weevils, termites) (Clifton et al. 2020). While EPF can have desirable effects on insect pests, some are also known to potentially impact on non-targets including vertebrates through the production of certain toxic biomolecules (Caloni et al. 2020; Zimmermann 2007).

Numerous EPF are known to affect Australia endemic insects. However, *S. frugiperda* is a recently established species, and the ability of regional EPF to infect this exotic species is unknown. There is a high probability that some local EPF that infect other lepidopteran species will also be pathogenic to *S. frugiperda*. There are currently approximately 900 Australian endemic and regional EPF isolates in the Commonwealth Scientific and Industrial Research Organisation (CSIRO) fungal collection (established by Dr Richard J. Milner). The present study is, therefore, the first step that aims to identify which EPF isolates will show promising impact towards *S. frugiperda* at various life stages (without progressing to bioassay until their safety on non-targets especially on vertebrates could be assessed), and to establish a standardised protocol for future laboratory assessments of other EPF isolates within the CSIRO collection.

## Materials and methods

### Fungal isolates

**Fig. 1:**
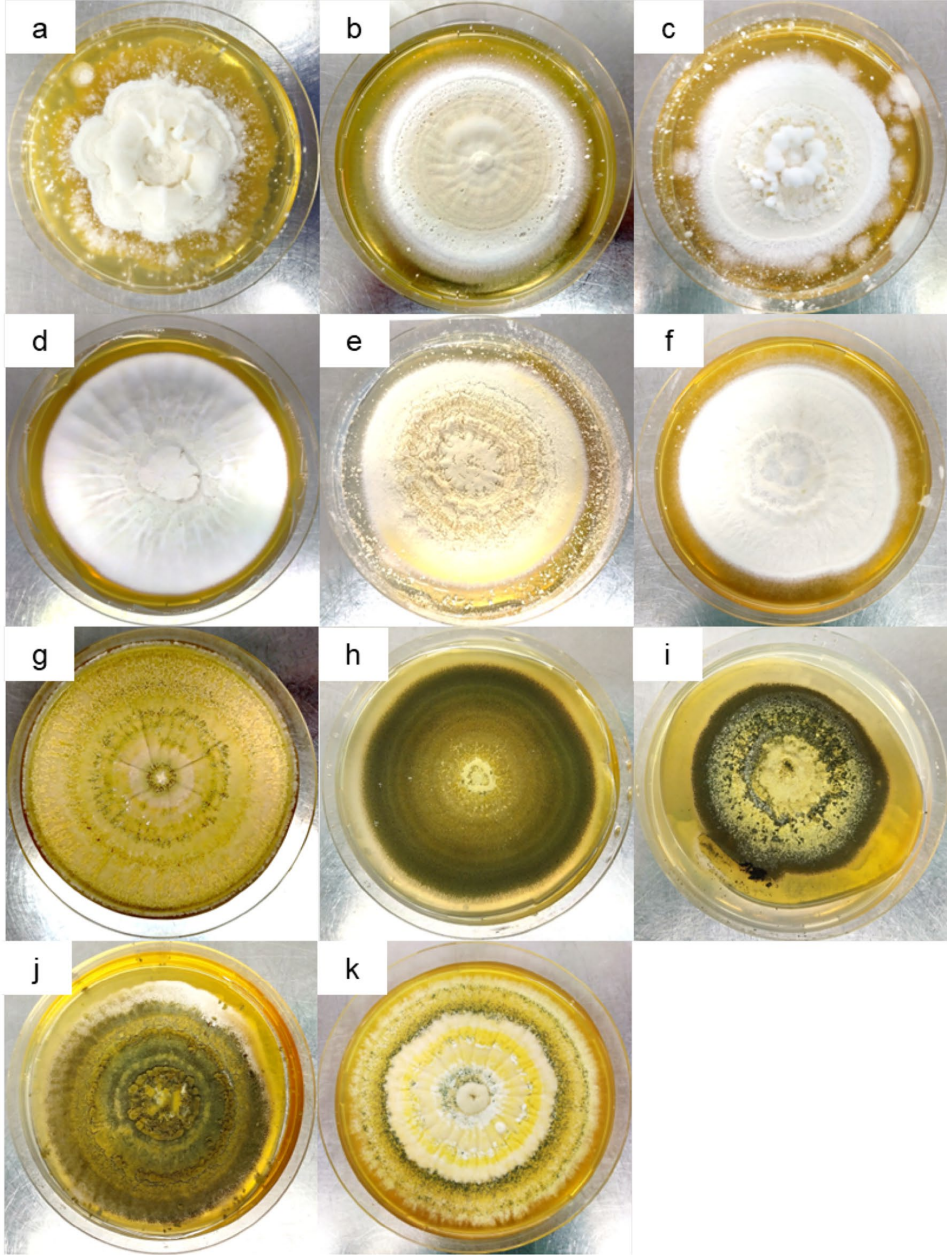
Morphology of the 11 fully grown fungal candidates at between 14 and 28-days post inoculation on individual SDAY media plates: **a** B-0016, **b** B-0077, **c** B-0079, **d** M-0121, **e** M-0122, **f** M-0123, **g** B-0571, **h** B-0698, **i** M-0999, **j** M-1000, and **k** B-1311.

Eleven fungal isolates were obtained from the CSIRO fungal collection (Black Mountain Laboratories, Canberra, Australia). These included six isolates of *Beauveria* sp. (B-0016, B-0077, B-0079, B-0571, B-0698 and B-1311) and five isolates of *Metarhizium* sp. (M-0121-0123 and M-0999-1000) that originated from lepidopteran hosts (Table 1, Fig. 1). The spores of these isolates had been freeze-dried and preserved at –80 °C. The fungi were revived and cultured on Sabouraud dextrose agar media with 1% yeast extract (SDAY; pH 5.6) and incubated at 28 ± 1 °C, 50 ± 10% relative humidity and under dark conditions.

**Table 1:** The fungal isolate code, fungal species, lepidopteran host species, geographic origin, and collection date of the fungal isolates from the CSIRO Black Mountain fungal collection.

Isolate code	Species	Host species	Geographic origin	Collection date
B-0016	*Beauveria* sp.	*Oncopera alboguttata*	Ebor, NSW, Australia	13/02/1980
B-0077	*Beauveria* sp.	Australian native budworm (*Helicoverpa punctigera*)	Geraldton, WA, Australia	18/09/1984
B-0079	*Beauveria* sp.	Monarch butterfly (*Danaus plexippus*)	QLD, Australia	11/10/1984
B-0571	*Beauveria* sp.	*Helicoverpa* sp.	Lucerne, Gatton, QLD, Australia	24/01/1990
B-0698	*Beauveria* sp.	*Oxycanus* sp.	Canterbury Plains, SI, New Zealand	8/10/1990
B-1311	*Beauveria* sp.	Emperor gum moth (*Opodiphthera eucalypti*)	Dorrigo, NSW, Australia	10/03/1999
M-0121	*Metarhizium* sp.	*Oncopera alboguttata*	Ebor, NSW, Australia	17/12/1985
M-0122	*Metarhizium* sp.	*Oncopera alboguttata*	Ebor, NSW, Australia	17/12/1985
M-0123	*Metarhizium* sp.	*Oncopera alboguttata*	Ebor, NSW, Australia	15/01/1986
M-0999	*Metarhizium* sp.	*Spodoptera* sp*.*	Cameron Highland, Pahang, Malaysia	11/05/1993
M-1000	*Metarhizium* sp.	*Spodoptera* sp.	Cameron Highland, Pahang, Malaysia	11/05/1993

### Fungal identification

To extract fungal genomic DNA (gDNA) for species identification, a mass of the fungal colony was collected from the SDAY plate and transferred into 300 μl cell lysis buffer. Following this, 20 μl of proteinase K (20 mg/ml, JetFlex™, A30701) was added and incubated at 58 °C for 16–20 h overnight. The rest of the DNA extraction process was performed using a JetFlex™ Genomic DNA Purification Kit (ThermoFisher, A30701) following the manufacturer’s tissue extraction protocol. gDNA was re-suspended by 30 μl adding TE buffer and incubated at 25 ± 1 ℃ for 16–20 h overnight. The purity and quantification of gDNA results were assessed using gel electrophoresis and on the Qubit 2.0 fluorometer (Life Technologies Corporation). The extracted DNA from each isolate was then stored at –20 °C until needed.

To determine genera/species of fungal candidates, the ITS1-5.8-ITS2 region of the nuclear ribosomal DNA (rDNA) was amplified via polymerase chain reactions (PCR) using universal primers ITS1 (5′TCCGTAGGTGAACCTGCGG) and ITS4 (5′TCCTCCGCTTATTGATATGC) (Raja et al. 2017). The PCRs were conducted using Platinum™ *Taq* DNA Polymerase kits (ThermoFisher, 10,966,018), 10 mM dNTP’s set (ThermoFisher, 10,297,117), 0.2 µM of each universal ITS1 and ITS4 primers, 2 mM of MgCl_2,_ and 2 µL of extracted genomic DNA, made up to a final 50 µL PCR volume with UltraPure™ Dnase/Rnase-Free Distilled Water (ThermoFisher, 10,977,015). The PCR reaction was prepared by following Platinum™ Taq DNA Polymerase protocols. A temperature gradient PCR was conducted following the protocol described by Oliveira et al. (2012). This began with an initial denaturation step at 94 °C for 3 min, followed by 35 cycles of 30 s at 94 °C, then 30 s at 55 °C for the annealing process, the template extension step at 72 °C for 1 min, and a final 10-min extension cycle at 72 °C. The PCR was carried out on a C1000 Touch™ Thermal Cycler (BIO-RAD).

The purification used 10 µL of the PCR product and followed the manufacturer’s instructions using a QIAquick PCR purification kit (Qiagen, 28,106). The purified PCR product was diluted with 30 µL of UltraPure™ Dnase/Rnase-Free Distilled Water (ThermoFisher, 10,977,015) and stored at −20 °C. For Sanger sequencing, PCR amplicons were sent to the Biomolecular Resource Facility at John Curtin School of Medical Research, Australian National University (ANU), Canberra, Australia.

The fungal ITS sequences analyzed were edited using pregap and Gap4 within the Staden package 2.0.0b11-2016 (Staden et al. 1999) and Geneious Prime 2021.2 (Biomatters Inc., Auckland, New Zealand). Sequences were aligned using MUSCLE (multiple sequence comparison by log-expectation) 3.8.425 (Edgar 2004) within Geneious based on default parameters (group sequences by similarity with eight iterations) prior to trimming to 538 bp (*Beauveria* spp.) and 534 bp (537 bp with gaps) (*Metarhizium* spp.). Sequences were identified by homology using Basic Local Alignment Search Tool (BLAST; https://blast.ncbi.nlm.nih.gov/Blast.cgi) via Geneious (2022.1.1) with default settings (NCBI Nucleotide collection with Megablast). The species of each isolate was tentatively identified based on the likelihood (% nucleotide identity) against the reported reference sequences from GenBank.

The maximum-likelihood (ML) phylogenetic trees of the CSIRO *Beauveria* and *Metarhizium* isolates were aligned using MAFFT (Katoh and Standley 2013) within Geneious v11.1.5 and gaps were visually re-aligned where necessary prior to being constructed using IQ-Tree (Trifinopoulos et al. 2016) with automatic evolutionary model selection, and node confidence estimated using 1000 bootstrap resamplings via UF-Boot (Minh et al. 2013). The ITS sequences of *Beauveria* spp. and *Metarhizium* spp. for comparison were obtained from the NCBI GenBank DNA database (Table 2) with the sequences having ≥ 97% identical sites (following the number of identical sites in previous studies; Izzo et al. 2005; Smith et al. 2007; Ryberg et al. 2008; Giusti et al. 2021) to the CSIRO fungal isolates. Visually re-aligned ITS sequences of *Beauveria* and *Metarhizium* spp. are provided as Suppl. Data 1 and 2, respectively. IQ-Tree output consensus tree files were exported in Newick format for manually manipulation and presentation using Dendroscope (Huson et al. 2007)**.**

**Table 2:** Reference ITS sequences from related *Beauveria* and *Metarhizium* species and their respective GenBank accession numbers used in the PhyML phylogenetic analyses

NCBI Accession number	Species	References
MN833071	*B. felina*	(Ramanujam et al. 2021)
OL375169	*B. bassiana*	(Rajula et al. 2021)
OL375165	*B. bassiana*	
OL375168	*B. bassiana*	
OL375170	*B. bassiana*	
OL375173	*B. bassiana*	
OL375167	*B. bassiana*	
JF837121	*B. bassiana*	(Ramanujam et al. 2020)
MN602591	*M. rileyi*	
MN907775	*M. rileyi*	
JF837146	*M. anisopliae*	
JF837154	*M. anisopliae*	
KJ728726	*N. rileyi*	(Visalakshi et al. 2020)
KJ728725	*N. rileyi*	
MH860365	*M. rileyi*	
JQ686668	*N. rileyi*	
KY436756	*M. rileyi*	
MF681620	*M. robertsii*	(Hernandez-Trejo et al. 1970)

### Insect samples

The research was conducted using lab colonies of two pest noctuids: the recently established *S. frugiperda* and the endemic* Helicoverpa armigera* Hübner (subspecies ‘*conferta’*; Hardwick 1965; Anderson et al. 2016; Pearce et al. 2017; Zhang et al. 2022; hereafter as ‘*H. armigera*’). The *S. frugiperda* colony was initially collected from Walkamin, Queensland, Australia (Tay et al. 2022a, b), while the *H. armigera* colony was originally established from cotton fields in the Namoi Valley, northern NSW Australia. Both have been reared at CSIRO in Canberra from 6th March 2020 (*S. frugiperda*) and the mid-1980s (*H. armigera*). We included *H. armigera* in the bioassay experiments to determine the viability and pathogenicity of these fungal isolates (some of which were isolated directly from *Helicoverpa* spp. and had been kept as freeze-dried spores since 1980 to 1999; Table 1), as well as to enable direct comparisons of bioassay protocols, baseline susceptibility, and future in-filed application of these candidate EPF against the endemic broadacre *Helicoverpa* pest species (i.e., *H. punctigera*, *H. armigera*), similar to the trailed insecticide and Bt bioassay experimental designs for Australia populations of FAW (Tay et al. 2022a).

Fertilised eggs were collected and transferred to a plastic bag and allowed to hatch overnight under standard rearing conditions (25 ± 1 ℃, 50 ± 10% relative humidity, and 14:10 day-night lighting). After 24–48 h, 1st instar caterpillars were gently placed into 45-well plates filled with the respective *S. frugiperda* (see Suppl. Data 1) and *H. armigera* artificial diet (Apirajkamol et al. 2020). Initial trail bioassay experiments using selected fungal isolates B-0571 and B-1311 on neonates and 1st instar larvae from both *S. frugiperda* and *H. armigera* showed that both could not survive submersion in the control (i.e., 0.1% Tween 80 and water without the entomopathogenic fungal spores) and in spore suspension.

Once the caterpillars reached the 3rd instar stage, they were transferred into 32-well plates filled with artificial diet specific for *S. frugiperda* (Tay et al. 2022a) or for *H. armigera* (Apirajkamol et al. 2020). Upon pupation, 40 pupae were randomly selected and transferred to a moth rearing basket that contained a honey solution (Apirajkamol et al. 2020) for nutrition. After approximately two weeks, the moths emerged, mated, and began to lay fertilised eggs. To maintain the population, eggs were regularly collected and reared through to adult following the steps described above.

### Spore collecting, concentration, and viability

To collect fungal spores, 10 ml of 0.1(v/v) % Tween 80^®^ solution was poured onto a culture plate and the surface of the fungal colony gently scraped to release the spores. The spore suspension was collected using a single channel automatic micropipette and stored at 4 °C for 3–5 days. The spore concentration was estimated using Haemocytometer (Bright-Line^®^, Z359629) and a light microscope (Olympus, CX40-PH).

Spore viability was analyzed following a protocol modified from (Tupe et al. 2017). The spore suspensions were diluted into 1–3 × 10^–3^ spore/ml concentration with 0.1%(v/v) Tween 80^®^ solution, then the diluted spore suspension was spread (100 µL) onto SDAY media. After 2–5 days of incubation the number of colonies were counted, and spore viability was estimated. For the bioassay, a spore concentration at ≥ 10^7^ conidia/ml was chosen (following Tupe et al. 2017). In addition, the work of Mubeen et al. (2022) suggests that there is no statistically significant mortality difference for fungal spore concentrations of 10^7^–10^9^ conidia/ml in *S. frugiperda*. We also ensured that the spore viabilities of all candidates were ≥ 85%. To generate reliable data, the spore count and viability was performed in three technical replications.

### Insect bioassays

The development of study samples was closely monitored and randomly collected for use in the bioassay experiments upon reaching early-3rd instar, 6th instar caterpillars, pupae (within three days of pupation), and adults (within a day of emergence).

Third instar *S. frugiperda* and *H. armigera* grow at variable rates, which could lead to inconsistent results if insects varied in size between treatments and if there is an association between size and susceptibility to pathogens. To check for size differences in samples of different treatments, a quarter of the samples in each treatment were randomly selected, weighed and compared.

To infect the study samples, 3rd instar caterpillars (*N* = 32), 6th instar caterpillars (*N* = 16), pupae (*N* = 16 unsexed), and adults (eight females and eight males) were submerged using feather-light forceps (Australian Entomological Supplies, E122B) for three seconds in 0.1% Tween 80^®^ (control) or fungal suspensions. Note that we did not disinfect the caterpillars prior to fungal bioassay treatments to minimise complications that could arise from disinfecting chemicals (e.g., potential impact to survival of fungal spores from the disinfectant). Infected samples were placed into 32-well plates filled with artificial diet for caterpillars and clean 32-well plate for pupae. Adults were transferred to a cylinder-shaped plastic container with a honey solution provided to allow them to mate (one pair per container). Infected and control samples were then reared under standard insect rearing conditions (see above) for 7–14 days. Mortality of the treated and control samples was recorded daily for caterpillars and moths, and two weeks following infection for pupae. Overall fecundity, and fertility of treated and control adult *S. frugiperda* was also recorded daily following infection.

### Statistical analysis

To estimate overall mortality caused by the candidate EPF treatments, the number of dead insects in treated samples was subtracted from the number of dead insects in respective control samples. While the cumulative and daily mortality are presented as is. The bioassay experiments were conducted with at least three biological replications, and the means were statistically compared.

Overall and daily mortality were statistically analysed using R version 4.1.0 (released 18-5-2021). One-way ANOVA was used to identify group differences, and the Tukey HSD (Honestly Significant Difference) post-hoc test with a 95% confidence interval was used to determine significant differences between treatments while taking into account the ≥ 3 replications. Tukey’s HSD was chosen to reduce the possibility of accepting false null hypothesis (false negative) from a high standard deviation or rejecting a true null hypothesis (false positive) from a high familywise error rate because of the number of treatments.

The R Packages used to perform the analyses included tidyverse for data manipulation (Wickham 2011), TukeyC (Faria et al. 2014) for the Tukey HSD analysis and homogenous subset result output, ggplot2 (part of the tidyverse metapackage), and ggtext (Wickham 2011) for graph creation.

## Results

### Fungal isolate species identification

**Fig. 2:**
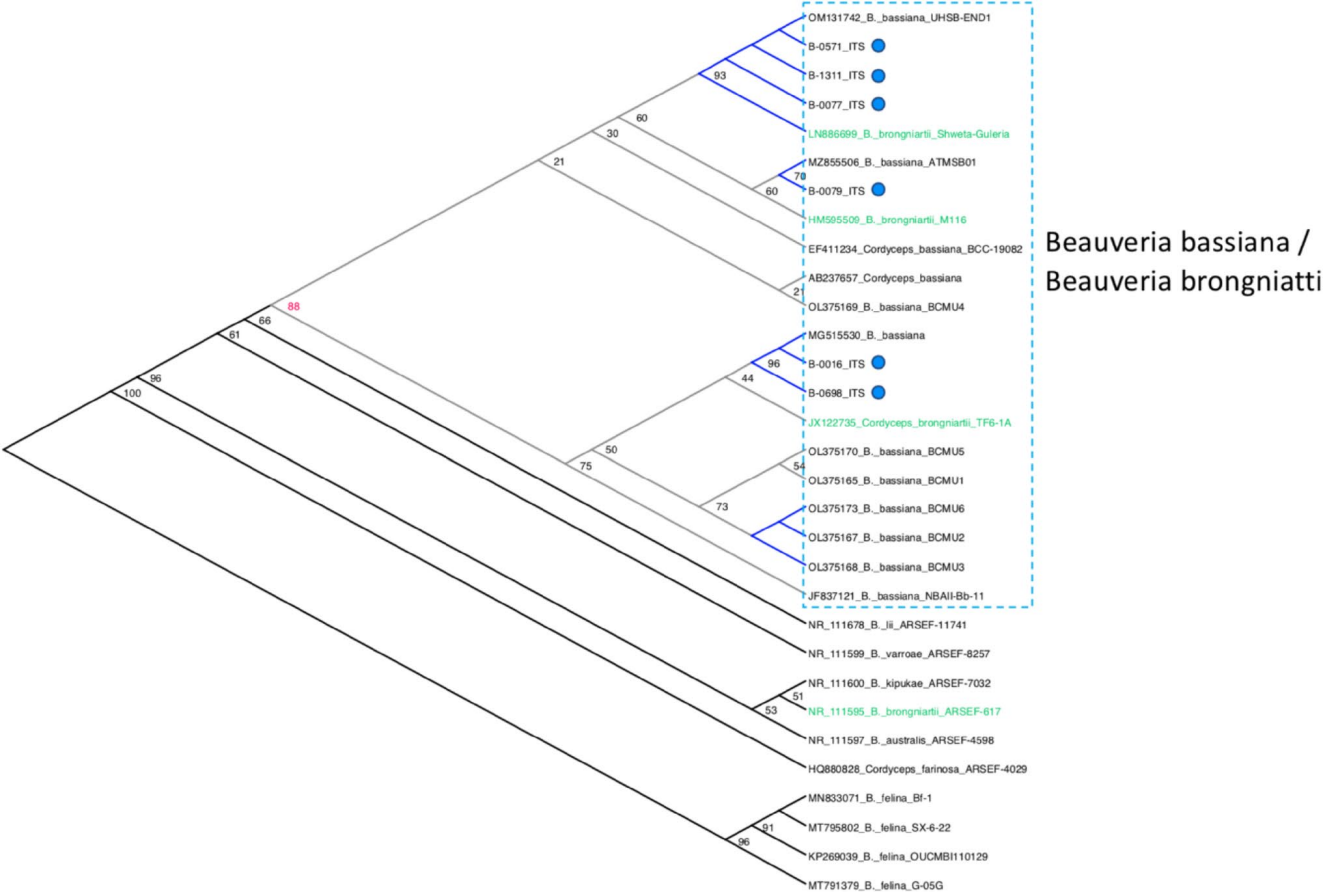
Maximum Likelihood (ML) phylogenetic inference of *Beauveria* and *Cordyceps* species including the six CSIRO *Beauveria* isolates (B-0016 (OM865408), B-0077 (OM865405), B-0079 (OM865406), B-0571 (OM865404), B-0698 (OM865407), and B-1311 (OM865403) using IQ-Tree with node support estimated from 1000 UF-Boot replications. The *Beauveria* isolates clustered with high confidence (88% node support) with various *B. bassiana*/*B. brongniatti* partial ITS sequences, although the phylogenetic position of *B. brongniatti* is less certain with an isolate (NR_111595) being placed outside of this *B. bassiana*/*B. brongniatti* clade. CSIRO *Beauveria* isolates are indicated by blue circle. Blue coloured branches within individual subclades shared 100% nucleotide identity. Differences in efficacies of causing mortality to the target pest *S. frugiperda* between the *Beauveria* isolates suggested that these might represent different strains. Refer to main text

The ITS sequence results suggest that the six candidate *Beauveria* fungal isolates (B-0016, B-0077, B-0079, B-0571, B-0698, and B-1311) had a high percentage of nucleotide identity (97–100%) to the many *Beauveria* spp. reference sequences. The six CSIRO *Beauveria* isolates were clustered with both *B. bassiana* and *B. brongniarti* with strong bootstrap support (88%) based on ITS sequence phylogeny (Fig. 2) as indicated by blue dashed-line box (note that *Cordyceps bassiana* and *C. brongniartii* are anamorphs of *B. bassiana* (Sung et al. 2006) and *B. brongniartii* (Shimazu et al. 1998; Sasaki et al. 2007), respectively). The phylogenetic position of *B. brongniattii* is also less certain with NR_111595 being found outside of this main clade (Fig. 2).

**Fig. 3:**
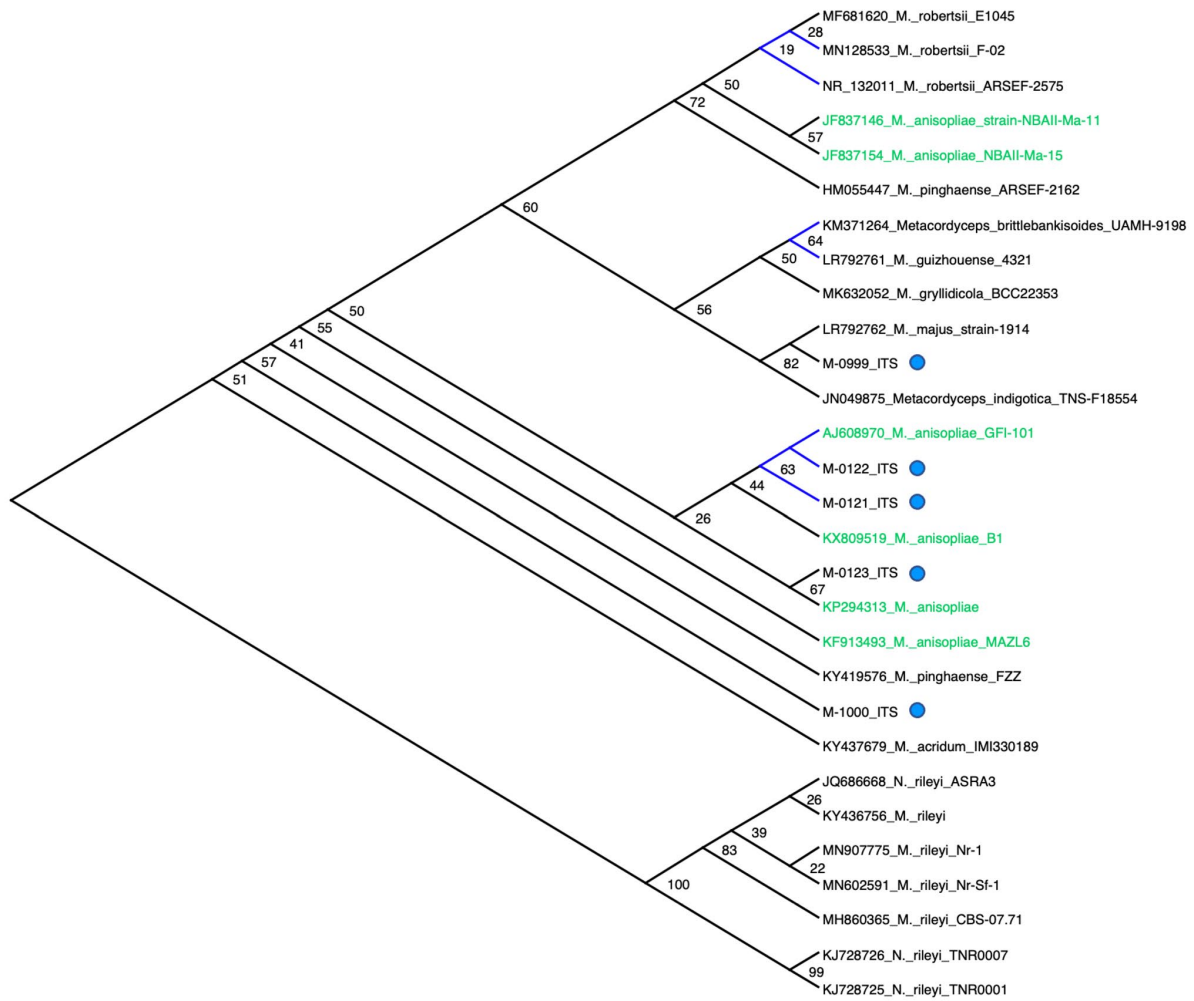
Phylogenetic placements of CSIRO’s *Metarhizium* isolates (indicated by blue circles) used in the bioassay experiment against *S. frugiperda*. Phylogeny inference was based on 630 bp sequences of the 18S ribosomal RNA partial gene consisted of the ITS1-5.8S-ITS2 gene region. Branches in blue within individual sub-clades represent samples that shared 100% nucleotide identity. Phylogenetic placements and species identity of the CSIRO isolates (GenBank accession numbers: OM865411–OM865413; OM865409–OM865410) were poorly defined due to low node support values. The overall poor phylogenetic placement of the *Metarhizium*/*Numuraea* species based this 630 bp of partial 18S rRNA gene is evident, as shown by the inconsistent phylogenetic positions of *Metarhizium anisopliae* isolates (shown in green)

The ITS sequences of the five CSIRO *Metarhizium* isolates (M-0121-123 and M-0999-1000) matched GenBank deposited ITS sequences (97–100% identical sites) of various *Metarhizium* and *Metacordyceps* species. The phylogenetic tree constructed with ITS sequences from the five CSIRO *Metarhizium* isolates suggests that M-0121 and M-0122 are closely related (both isolates have identical ITS nucleotide identity), and together with M-0123, loosely clustered with three *M. anisopliae* (AJ608970, KX809519, KP294313). The isolate M-0999 clustered with *M. majus* (LR792762) and *Metacordyceps indigotica* (JN049875; anamorph of *M. indigoticum*; Kepler et al. 2012, 2014) with a relatively high node support value (82%). The isolate M-1000 appeared to be unique and having a phylogenetic position away from the branches that have the various *M. anisopliae* ITS sequences. Interestingly, *Metacordyceps brittlebankisoides* (KM371264, anamorph of *M. brittlebankisoides*; Kepler et al. 2012)*,* and *M. guizhouense* (LR792761) appeared to share the same ITS sequence identity as indicated by the blue coloured branches (Fig. 3; Suppl. Data 2), highlighting a need for taxonomic revision and the limitations of molecular taxonomy based on the widely applied ITS gene marker. The GenBank accession numbers of the CSIRO *Beauveria* and *Metarhizium* ITS sequences are OM865403–OM865413.

### Mortality of *Spodoptera frugiperda* 3rd instar caterpillars

A subsample of the population used in bioassays was weighed to compare their development and the results suggest that there is no significant difference in size among the samples (F_71,504_ = 1.30, *P* = 0.062).

**Fig. 4:**
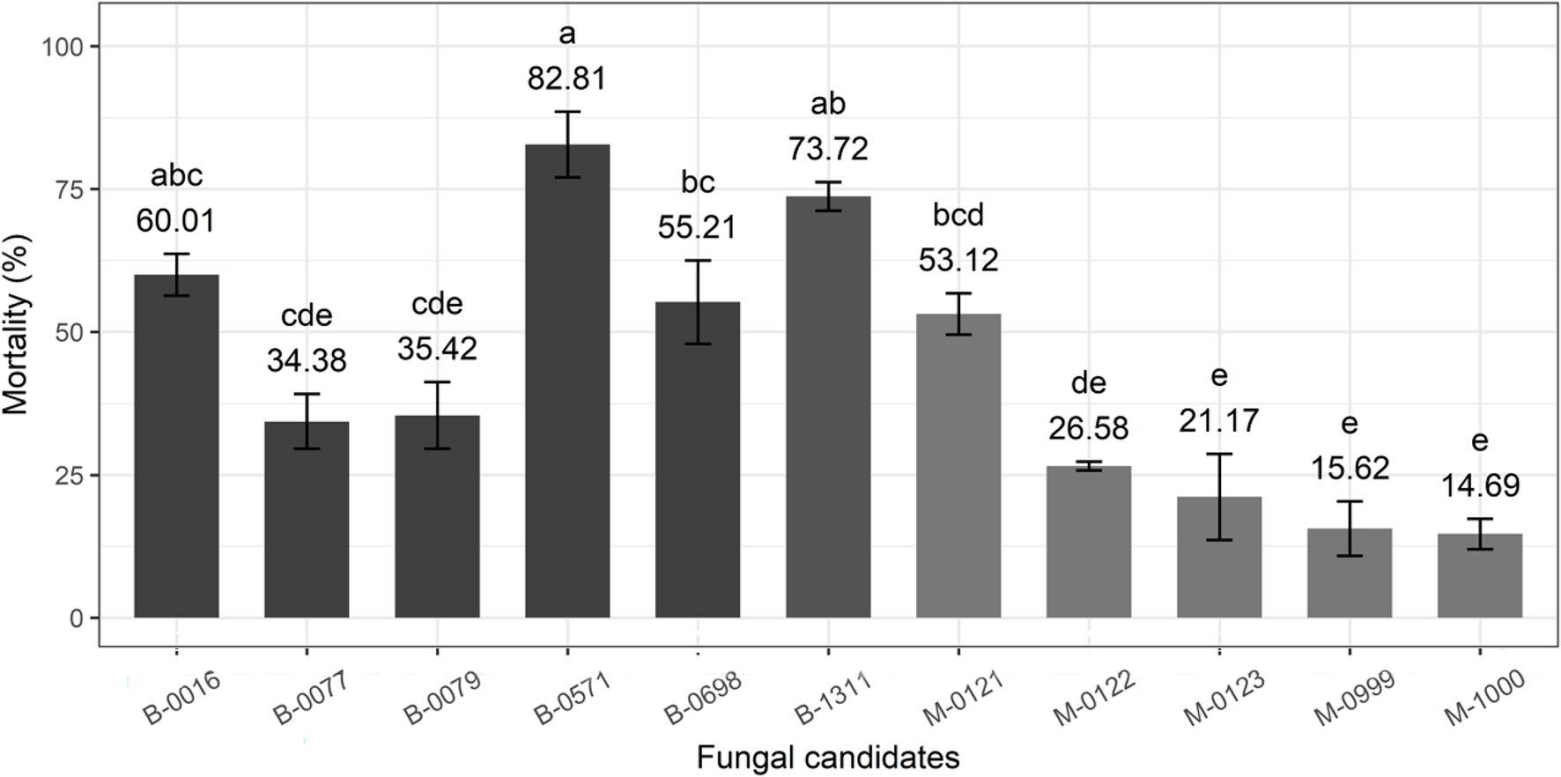
Overall mortality (%) of 3rd instar *S. frugiperda* caterpillars seven days after infection with the fungal candidates. Bars sharing letters are not significantly different. The error bars show standard error. Bars with darker shades of grey represent samples treated with *Beauveria* while the lighter grey bars represent samples treated with *Metarhizium*

Seven days following infection, three isolates (B-0016, B-0571, and B-1311) of *Beauveria* sp. induced the highest overall mortality in 3rd instar caterpillars (F_10,28_ = 25.51, *P* < 0.001, 60.01 ± 3.66, 82.81 ± 5.75, and 73.72 ± 2.51 respectively, Fig. 4), followed by the *Metarhizium* isolate M-0121 (53.13 ± 3.61%) and the *Beauveria* isolate B-0698 (55.21 ± 7.29%). Mortality induced by the two remaining *Beauveria* isolates (B-0077 and B-0079) and four *Metarhizium* isolates (M-0122, M-0123, M-0999, and M-1000) was significantly lower (Fig. 4).

**Fig. 5:**
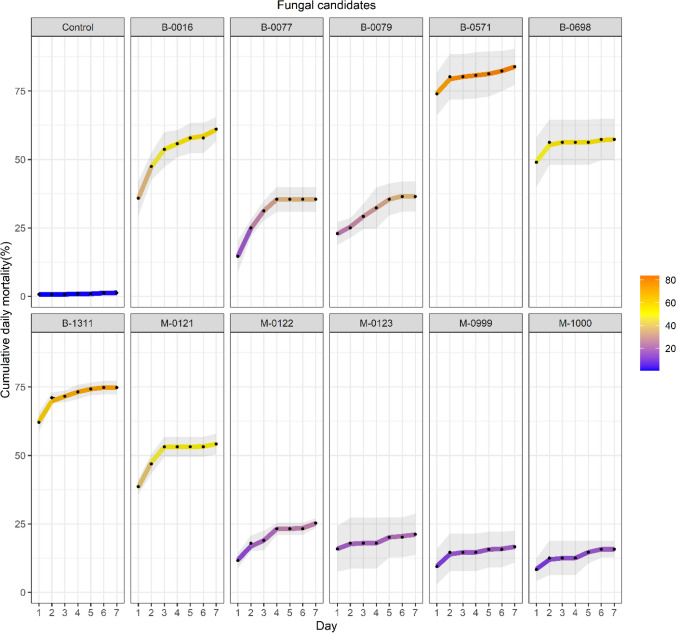
The cumulative daily mortality (%) of 3rd instar *S. frugiperda* infected with eleven EPF candidates. Third instar *S. frugiperda* were treated with a spore suspension of EPF candidates or 0.1%(v/v) Tween 80^®^ solution for the control samples (*N* = 32 caterpillars with ≥ three replications). Mortality of treated samples was recorded daily for seven days following infection. The grey area represents the standard error while the black dot and the colour gradient line represent result means throughout the seven days of the experiment

Daily mortality and cumulative daily mortality after infection were recorded to assess killing speed. The results suggest that mortality in 3^rd^ instar *S. frugiperda* caterpillars treated with B-0571, B-0698, B-1311, M-0123, M-0999, and M-1000 occurred predominantly within the first two days following infection, particularly on the first day (Fig. 5 and Suppl. Data 3 Figures S1a, and S1b). The other EPF candidates (i.e., B-0016, B-0077, B-0079, M-0121, and M-0122) induced mortality more gradually from day one to day seven.

**Fig. 6:**
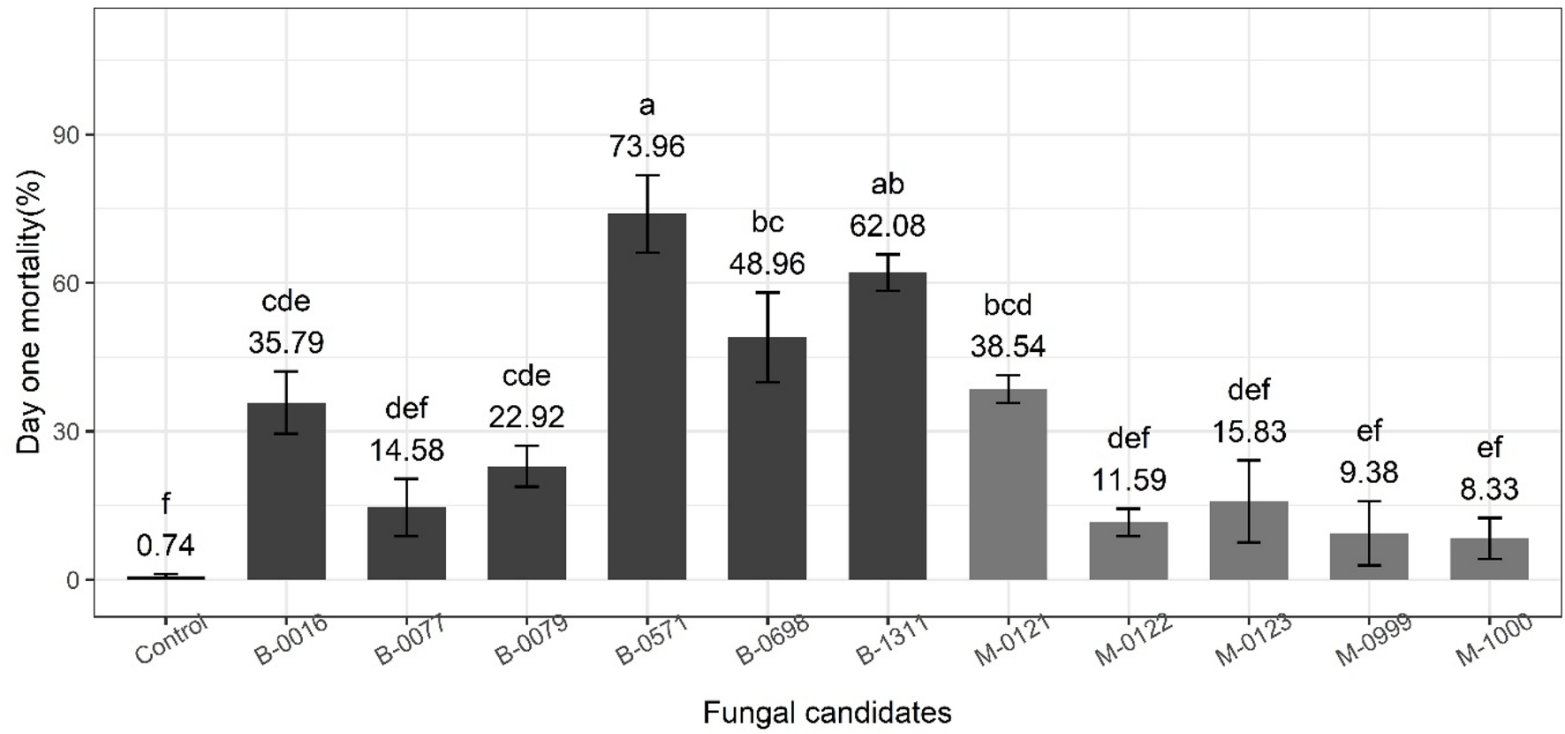
Day one mortality of 3rd instar *S. frugiperda* at 24 h after infection with EPF candidates. Bars sharing letters are not significantly different. The error bars show standard error. Bars with darker shades of grey represent samples treated with *Beauveria* while the lighter grey bars represent samples treated with *Metarhizium*

Because the first day is when the most deaths occur as compared with the control treatment, results were statistically analysed to ascertain which isolates induced the highest day one mortality (mortality within 24 h of infection). Samples infected with B-0571 and B-1311 had the highest day one mortality following infection (73.96 ± 7.85% and 62.08 ± 3.67%, F_11,44_ = 36.23, *P* < 0.001, Fig. 6), followed by B-0698 (48.96 ± 9.08%) and M-0121 (38.54 ± 2.75%). Although B-0016 treated samples are one of the three that have the highest overall mortality after seven days, its day one mortality is significantly lower than B-0571 and B-1311 (35.79 ± 6.32%, statistical results mentioned above). Day one mortality induced by other fungal candidates was significantly lower than those five isolates, but not significantly different to each other, with the day one mortality of the control samples (first 24 h post 0.1% (v/v) Tween 80^®^ application) being the lowest of all (0.74 ± 0.33%).

### Mortality of *Spodoptera frugiperda* 6th instar caterpillars, pupae, and adults

**Fig. 7:**
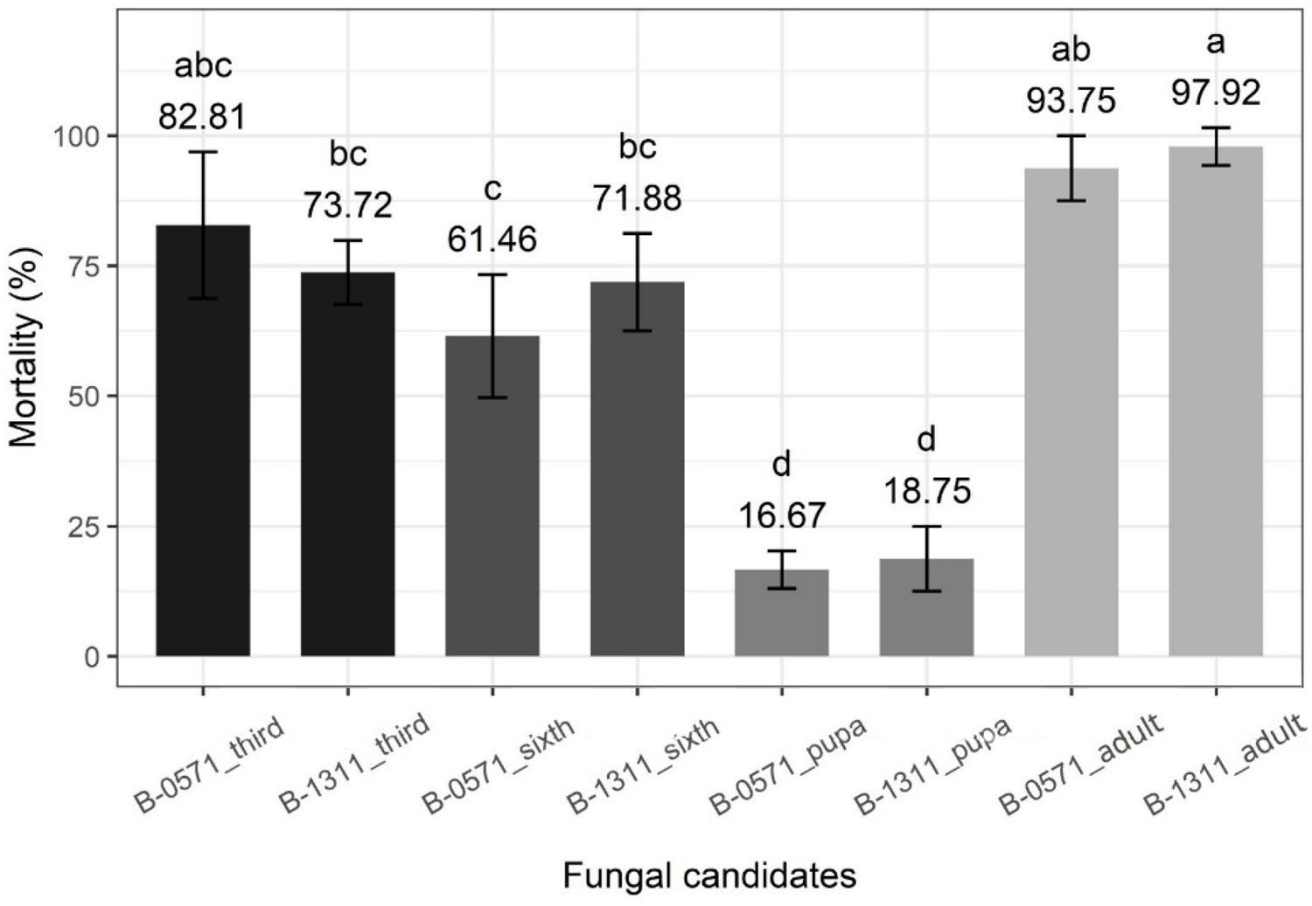
Overall mortality of *S. frugiperda* 3rd and 6th instar caterpillars, pupae, and adults treated with *Beauveria* sp. isolates B-0571 and B-1311. Bars sharing letters are not significantly different. The error bars show standard error. Bars with darker shades of grey represent samples. The life stages of treated samples are indicated in shades of grey

The effectiveness of the two most virulent strains of *Beauveria* sp. against 3rd instar *S. frugiperda* caterpillars (B-0571 and B-1311) was assessed against 6^th^ instar caterpillars, pupae, and *S. frugiperda* adults. B-0571 and B-1311 induced overall mortality of 61.46 ± 6.83% and 71.88 ± 5.41%, respectively, in 6th instar *S. frugiperda* caterpillars seven days following infection (F_1,4_ = 1.429, *P* = 0.298); 16.67 ± 2.08% and 18.75 ± 3.61%, respectively, in pupae (at day 14) (F_1, 4_ = 0.25, *P* = 0.643); and 93.75 ± 3.61% and 97.92 ± 2.08% respectively in moths (at day seven) (F_1, 4_ = 1, *P* = 0.374) (Fig. 7).

**Fig. 8:**
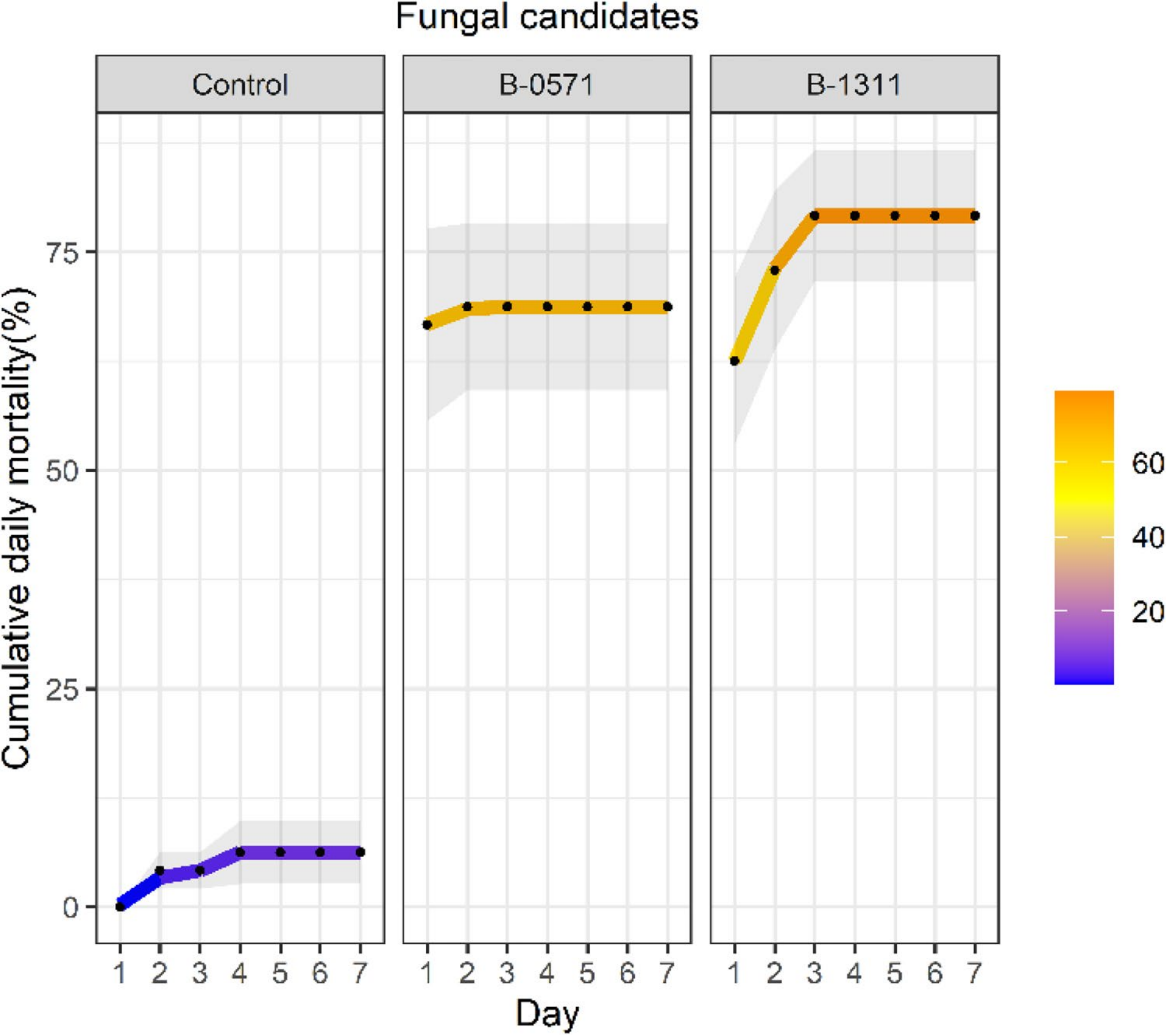
Cumulative daily mortality (%) of 6^th^ instar *S. frugiperda* caterpillars infected with *Beauveria* sp. isolated B-0571 and B-1311. Experiments were conducted with three biological replications and the results show the average daily mortality shown as dots and gradient line. Standard error is indicated as the grey areas behind dots and lines

**Fig. 9:**
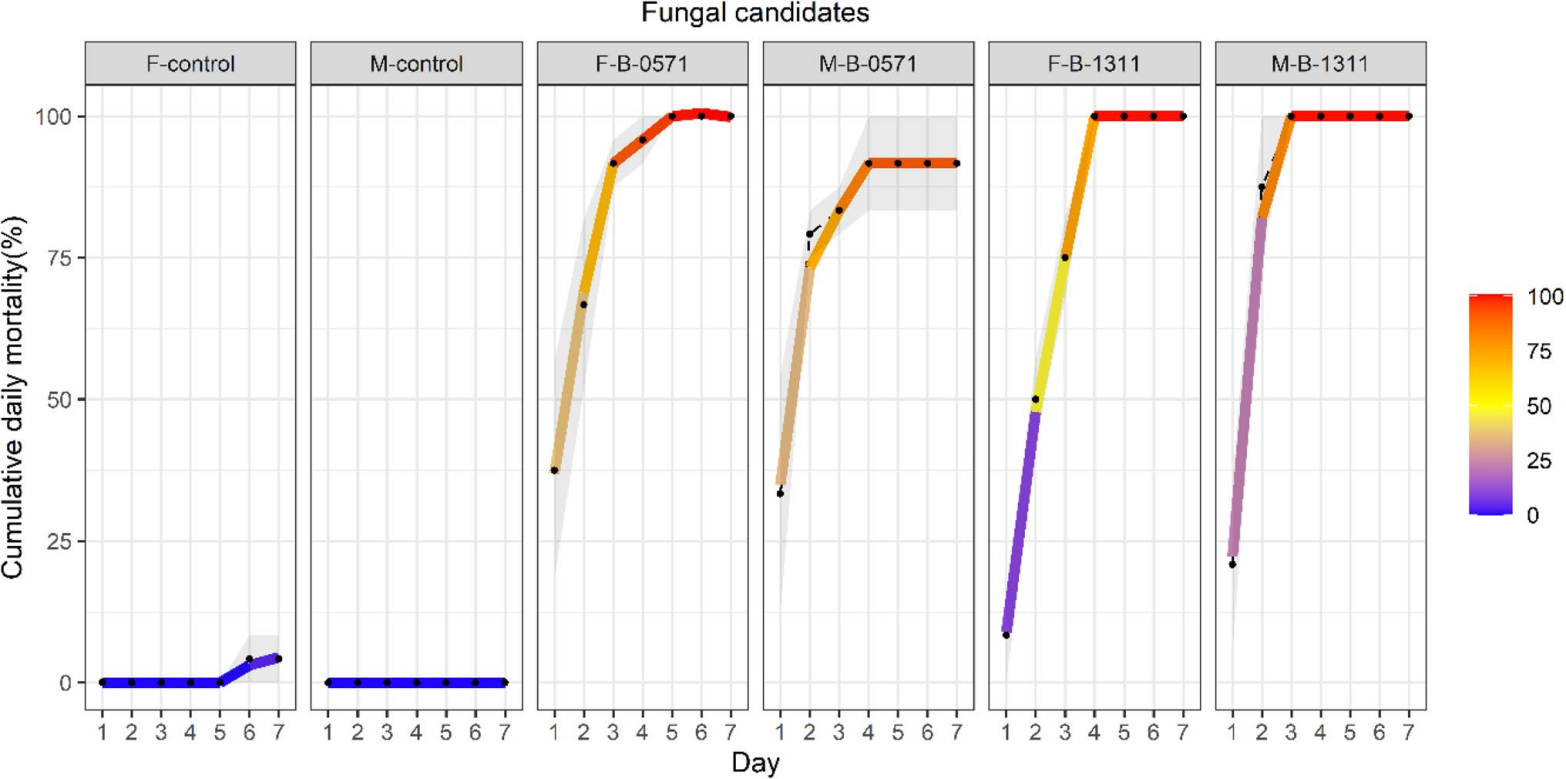
Cumulative daily mortality (%) of adult *S. frugiperda* infected with *Beauveria* sp. Isolates B-0571 and B-1311. Average daily mortality is illustrated as dots and gradient lines. The standard error is indicated as grey areas behind the gradient lines and the treatment and sex of study samples are specified as *F* female and *M* male

Daily mortality of 6th instar *S. frugiperda* infected with B-0571 and B-1311 was highest on the first day following infection (Control samples: 0%, B-0571: 66.67 ± 11.02% and B-1311: 62.5 ± 9.55%, Suppl. Data 3 Figure S2a). No deaths were observed after day two following infection in the B-0571 treated samples and after day five in the B-1311 treated samples (Fig. 8). Mortality at the adult stage mainly occurred within the first three days following infection (Fig. 9 and Suppl. Data 3, Figure S2b, cumulative mortality at day three: 91.67 ± 4.17% for B-0571 female, 83.33 ± 4.17 for B-0571% male, 75 ± 7.22% for B-1311 female, and 100 ± 0% for B-1311 male).

To determine the reproductive potential of infected adults, the number of females that produced eggs was recorded. Only 4.17 ± 1.47% of all treated adult females that were infected with B-0571 produced eggs, of which none hatched. None of the adult females that were infected with B-1311 laid any egg masses. As a result, none of the *S. frugiperda* infected with B-0571 and B-1311 isolates produced first-generation offspring. In contrast, 66.67 ± 1.47% of female control moths produced eggs and 75.56 ± 1.57% of these females produced offspring.

Comparing overall mortality across life stages at seven days after infection (F_7, 22_ = 39.01, *P* <  < 0.001, Fig. 7) highlights that both isolates B-0571 and B-1311 were particularly effective against *S. frugiperda*. Isolate B-0571 also induced high mortality in 3rd instar caterpillars that was not significantly different from the mortality in moths or 6th instar caterpillars. Mortality induced by isolate B-1311 in 3rd instar caterpillars, however, was significantly lower than in adults but was not significantly different from 6th instar caterpillars. For both B-0571 and B-1311, mortality was much lower in pupae than in other life stages.

### Mortality of 3rd instar *Spodoptera frugiperda* vs. *Helicoverpa armigera*

Weight of representative samples did not differ significantly across treatments and between *S. frugiperda* and *H. armigera* (F_17,126_ = 0.7812, *P* = 0.71), and as such any size- or development-associated effects can be excluded.

**Fig. 10:**
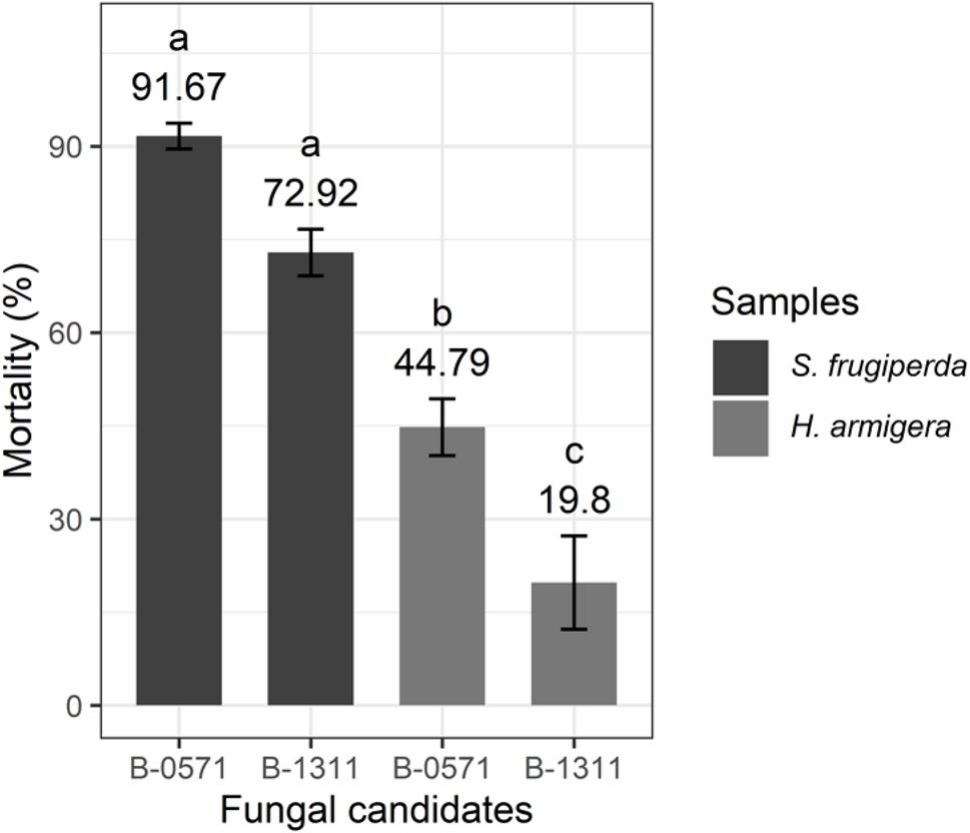
Overall mortality (%) of *S. frugiperda* and *H. armigera* infected with *Beauveria* sp. isolates B-0571 and B-1311 at day 14 following infection. Bars sharing letters are not significantly different. The error bars show standard error. Bars with darker shades of grey represent *S. frugiperda* while the lighter grey bars represent *H. armigera*

Overall mortality (%) of 3^rd^ instar *S. frugiperda* and *H. armigera* after being infected with B-0571 and B-1311 at day 14 suggested that both isolates induced significantly higher mortality in *S. frugiperda* than in *H. armigera* (F_3,8_ = 41.72, *P* < 0.001, Fig. 10). Isolate B-0571 (originally isolated from *Helicoverpa* sp., see Table 1) induced 91.67 ± 2.08% mortality in *S. frugiperda* but only 44.79 ± 4.54% in *H. armigera.* Similarly, isolate B-1311 (originally isolated from *Opodiphthera eucalypti*, Table 1) induced 72.92 ± 3.75% mortality in *S. frugiperda* but only 19.80 ± 7.51% in *H. armigera*.

**Fig. 11:**
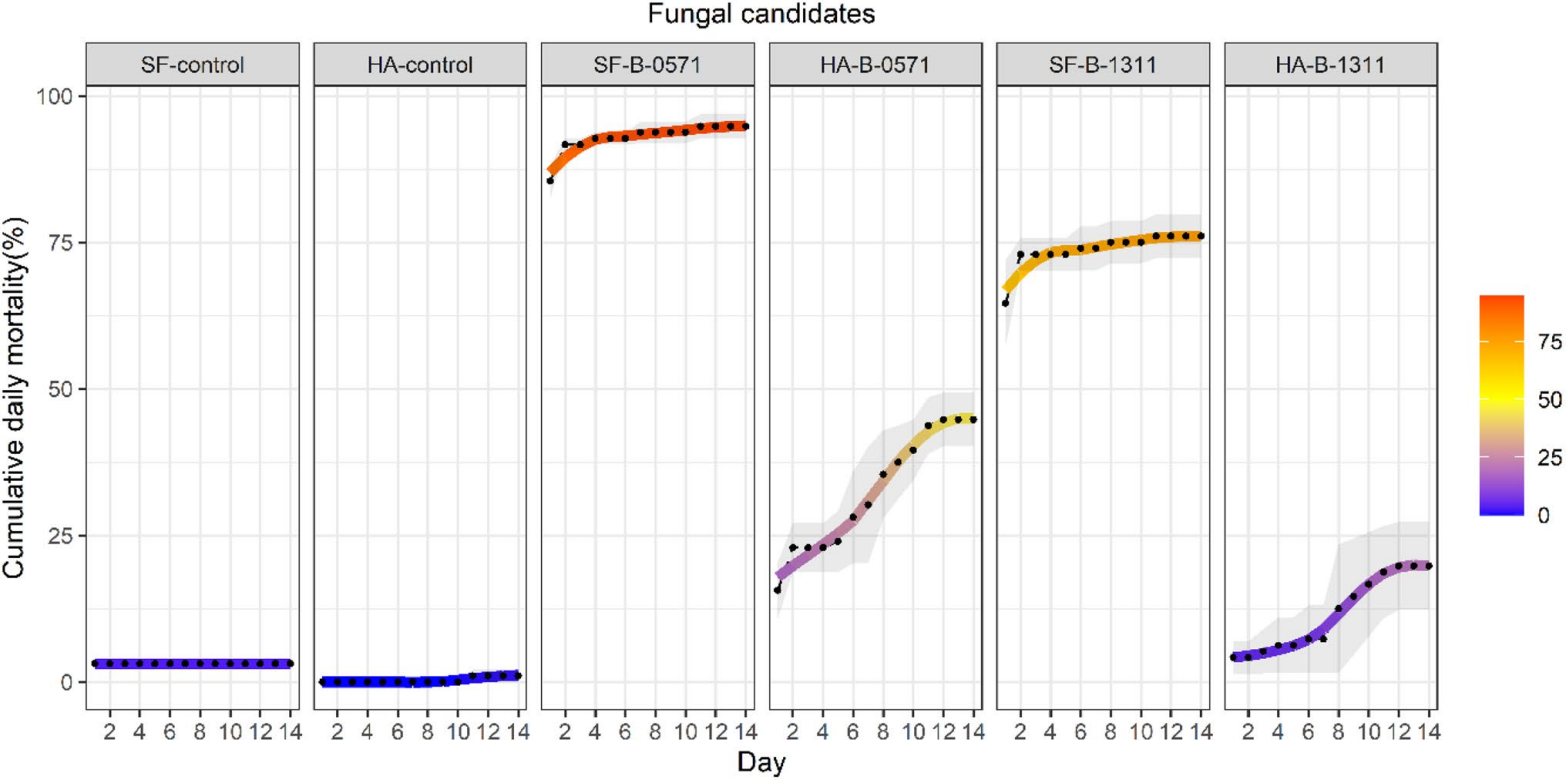
The cumulative daily mortality (%) of 3rd instar *S. frugiperda* and *H. armigera* infected with *Beauveria* sp. isolates B-0571 and B-1311 for 14 days following infection. The cumulative mean of each treatment in each day is represented by a black dot and the overall trend across 14 days is represented as a gradient line. The grey area represents the standard error

*Spodoptera frugiperda* caterpillars predominantly died within the first few days after infection. In contrast, while many of the treated *H. armigera* caterpillars died within two days after infection, mortality also progressed gradually throughout the 14-day period (Fig. 11 and Suppl. Data 3 Figure S3).

## Discussion

### Fungal isolate species identification

The candidate fungal species were identified via sequencing of the ribosomal 16S ITS region. Sequence results indicated that of the 11 fungal candidates, five *Beauveria* isolates matched the ITS of six species of *Beauveria* with high nucleotide identity (100–97%, B-0016, B-0077, B-0079, B-0571, B-0698, and B-1311), and five *Metarhizium* isolates matched the ITS regions of various *Metarhizium* species (M-0121, M-0122, M-0123, M-0999 and M-1000). The general consensus divergence for cut-off for delimitating across the Fungi kingdom is approximately 3–5% based on 500–800 bp of ITS sequences (intraspecific ITS variability in fungi is 2.51–4.57%; Schoch et al. 2012; Raja et al. 2017). In the phylum Ascomycota (which includes *Beauveria* and *Metarhizium*), the average variability in weight of infraspecific ITS has been reported to be 1.96–3.73% (Nilsson et al. 2008; Raja et al. 2017). Hence, although the genera of CSIRO fungal isolates were inferred by ITS sequences analysis (six isolates of *Beauveria* sp. and five isolates of *Metarhizium* sp.), the species of all candidates remain poorly defined. This is because the ITS regions of those isolates have ≥ 97% nucleotide identity to multiple species from the reference sequences.

Based on the ITS sequence phylogeny, six of the CSIRO *Beauveria* fungal isolates clustered with high confidence with two *Beauveria* species including *B. bassiana* and *B. brongniarti,* suggesting that resolving the taxonomic status of both *B. bassiana* and *B. brongniarti* could not rely solely on the ITS region. Similarly, the five CSIRO *Metarhizium* isolates were clustered in the same clade, albeit less confidently, with many species of *Metarhizium*. This is due to high nucleotide similarity in *Metarhizium* ITS sequences both within the *M. anisopliae* complex (*M. anisopliae, M. guizhouense, M. pingshaense, M. robertsii,* and *M. acridum*) and other *Metarhizium* spp. (*Metacordyceps indigotica, M. gryllidicola,* and *Metacordyceps brittlebankisoides*). There is currently insufficient data for molecular diagnostics of the various *Beauveria* and *Metarhizium* isolates based on the ITS regions, and multilocus sequence typing (MLST)/whole genome sequencing approach may offer greater resolution power to aid in the molecular taxonomy and assist with molecular diagnostics of these important fungal groups.

Phenotypic characters such as macro-and micro-morphology, production of certain chemicals, and host specificity are also used to help identify fungal species. These phenotypic characters, however, can be affected by external factors (e.g., growth conditions) and the strain of fungus (Bridge et al. 1993; Francisco et al. 2006; Fernandes et al. 2010; Sepúlveda et al. 2016). For example, *B. brongniarti* has been reported to mainly attack cockchafer (i.e., *Melolontha* spp.) and other coleopteran insects (Vestergaard et al. 2003; Shah and Pell 2003; Imoulan et al. 2017; Rohrlich et al. 2018). Nevertheless, Wu et al. (2019) demonstrate that *B. brongniartii* SB010 could infect lepidopteran species such as *S. litura*. Molecular diagnostics and the molecular taxonomic status of CSIRO *Beauveria* and *Metarhizium* isolates used in this study will require further investigation, either through the whole genome sequencing approach or via a MLST approach involving other widely used DNA markers (i.e., *TEF1α*, *RPB1*, *RPB2*, *β-tubulin*, *CaM*, *Bloc*) (Bischoff and Rehner 2009; Hernández and Guzman-Franco 2017; Dizkirici and Kalmer 2019; Hoang et al. 2019; Bustamante et al. 2019; Castro-Vásquez et al. 2021).

### Mortality of *Spodoptera frugiperda* 3rd instar caterpillars

We investigated the overall and daily mortality of 3rd instar caterpillars to determine efficacies of each fungal candidate at the concentration of ≥ 10^7^ spores, as suggested by work of Tupe et al. (2017); Mubeen et al. (2022). Two isolates of *Beauveria* sp., B-0571 and B-1311, induced the highest mortality. The higher mortality levels induced by B-0571 and B-1311 could be because of the strength of spore adhesion, spore gemination rates, the production of enzymes and/or secondary metabolites, and growth conditions that support their development (see below).

The strength of spore adhesion is one of the crucial factors that indicates the virulence of EPF. Spore adhesion is the very first stage of fungal infection, and mostly relies on kinetic mechanisms (e.g., hydrophobic and/or electrostatic) (Boucias and Pendland 1991). Some strains of EPF may employ carbohydrate substances or specific receptors/ligands to strengthen the adhesion force (Boucias et al. 1988). A weak adhesion strength could result in the spores being washed off from the surfaces of insects (Holder and Keyhani 2005) thus preventing them from infecting the host species (Wang and St Leger 2007). Herein, a diverse isolate of fungi may manifest different level of hydrophobicity and biochemistry. This may result in a fluctuation in virulence. The two most pathogenic isolates of *Beauveria* sp. (B-0571 and B-1311) may exhibit stronger spore adhesion strength than other tested fungal candidates.

Conidial germination rates are also frequently associated with the virulence of EPF (Heale et al. 1989; Yeo et al. 2003; Safavi et al. 2007; Tseng et al. 2014). After attaching to the insect cuticle, to infect the target insect the spores must germinate and form an appressorium to penetrate through the insect’s chitinous exoskeleton. A faster germination rate is speculated to not only quicken the killing process but also reduce the possibility of losing spores from the insect moving and moulting (Altre et al. 1999). Faria et al. (2015) found that fast-germination strains of *B. bassiana* exhibited higher virulence towards *S. frugiperda* than do slow-germination strains. In the present study, the two fungal isolates that induced the highest overall mortality (B-0571 and B-1311) also have the highest day one mortality, which suggests that these fungal strains may have higher germination rates. At this time, germination rates of the candidate fungi have not been measured and so the contribution of germination rate to the observed mortality patterns remains unknown.

The production of hydrolytic enzymes has also been proposed as one determinant of EPF virulence. The arthropod exoskeleton is composed of various compounds including chitin, lipid, protein, and phenolic compounds that act as a barrier to protect the insect from desiccation and entomopathogens (Petrisor and Stoian 2017). EPF secrete a wide range of cuticle-degradation enzymes to infect the insect (e.g., chitinase, protease, and lipase) (Cheong et al. 2016). Consequently, fungal candidates that possess higher virulence may have higher enzyme activities. Further investigation is also required to verify this hypothesis for our high-performing *Beauveria* candidates B-0571 and B-1311.

Growth conditions of the fungal candidates could play significant roles in fungal virulence. Culture media and growth temperature have been shown to greatly influence the strength of spore adhesion (Ibrahim et al. 2002; Rangel et al. 2008), production of cuticle-degrading enzyme protease (Butt et al. 1996; Safavi et al. 2007; Rangel et al. 2008), conidial gemination rates (Yeo et al. 2003; Safavi et al. 2007; Rangel et al. 2008), and production of secondary metabolites (Asai et al. 2012; VanderMolen et al. 2013). Therefore, some of those isolates which exhibit a low killing ability may require different culture media or/and growth conditions to improve virulence.

Some EPF have the ability to secrete secondary metabolites that could contribute to the outcome of fungal-insect interaction. This is because secondary metabolites could assist EPF to overcome insects’ immune systems and quicken mycosis (Zimmermann 2007). There is evidence that some strains of *B. bassiana* are able to produce host-specific secondary metabolites that can cause 50% mortality at very low concentrations (3.3 µg/g body wt; Bassiacridin, infected *Locusta migratoria*) (Quesada-Moraga and Vey 2004). Regarding the high mortality induced by B-0571 and B-1311, these strains may produce bioactive compounds that have insecticidal activities toward *S. frugiperda*. Understanding and being able to produce these compounds could open new avenues for controlling invasive pest species, however, further investigation is required to confirm these hypotheses.

It is also important to be aware of the safety perspectives of EPF, some secondary bioactive compounds such as Beauvericin are known to be toxic to vertebrates including humans (Caloni et al. 2020). Fungi of the genus *Beauveria* have also been shown to be able to infect immunocompromised patients (Henke et al. 2002). We urge caution that research into developing biopesticides should take these factors into consideration, especially when undertaking further testing using candidate EPF isolates against human cell lines/organoids or other vertebrates. In addition, adopting the whole genome sequencing and transcriptomic approaches could help to identify potential risks of exposure to such toxins and help guide decisions on their eventual field trails and/or commercialisation, prior to undertaking bioassay experiments.

### Mortality of *Spodoptera frugiperda* 6th instar caterpillars, pupae, and adults

Isolates B-0571 and B-1311 which induced the highest mortality in *S. frugiperda* 3rd instar caterpillars also induced high morality in adults and 6th instar caterpillars, but not in pupae. Differences in the susceptibility of *S. frugiperda* at different developmental and life stages may be linked to the strength of the insect’s immune system, thickness, profile, and availability of susceptible locations on the exoskeleton, and pathogen defences (see below).

A more developed immune system could enhance the resistance of *S. frugiperda* toward fungal infection. In the present study, 6th instar caterpillars generally were less susceptible to pathogens than were 3rd instar caterpillars. Similarly, 5th instar *S. frugiperda* caterpillars are less susceptible to nematode (*Steinernema feltiae*) than 1st and 3rd instar caterpillars (Fuxa et al. 1988). The increasing of resistance in older caterpillars is believed to be strongly associated with the development of their immune system. Haemocytes tend to be much more abundant in older caterpillars than in younger caterpillars (Carper et al. 2019). This suggests that caterpillars increase their immunometabolism as they progress through their developmental stages. The stronger immune system would imbue older *S. frugiperda* with greater resistance against EPF (and/or insecticides) leading to reduced susceptibility to infection (or reduced mortality from exposure to insecticides).

The physical aspects of *S. frugiperda* at each life stage could also contribute to the outcome of fungal infection. Regarding the mode of action of *Beauveria* species, the spores infect the target host by penetrating through its external exoskeleton. Thus, the insect’s cuticular thickness and profile could be important factors in preventing penetration. According to Wrońska et al. (2018), the cuticles of caterpillars and the thorax of moths are the most susceptible areas to be digested by proteases and lipase (commonly produced by many EPF) (Erlacher et al. 2006; Hussein et al. 2012; Dhawan and Joshi 2017). This makes caterpillars and moths highly vulnerable to fungal infection. Additionally, the spores of EPF generally accumulate around the spiracles, hairs, pores of the wax glands, eyes, antennal segment, and legs articulating membranes and germinate from those areas (Toledo et al. 2010). Hence, thinner/weaker exoskeletons and/or higher availability of susceptible areas potentially provides the fungi with greater chances to successfully infect host species.

*S. frugiperda* commonly pupate in the soil and are, therefore, likely to encounter entomopathogenic microbes more than are caterpillars and adults. Thus, pupae may have evolved defence mechanisms, such as melanisation of the exoskeleton, to defend against soil-borne entomopathogens. Melanisation is a process that the insect employs to enhance cuticle pigmentation, cuticular sclerotization, wound healing, and its innate immune system (Sugumaran and Barek 2016). The darkness of the insect cuticle (higher concentrated melanin indicator) could, in some cases, indicate immune status (Nappi et al. 1995; Reeson et al. 1998; Barnes and Siva-Jothy 2000). Melanisation is likely to be a key mechanism that protects pupae from fungal infection. In addition, pupal cells of some insect species (e.g., *Curculio caryae*) have been shown to possess the antimicrobial properties which can suppress the growth and germination of *B. bassiana* (Shapiro-Ilan and Mizell 2015). Whether *S. frugiperda* also evolved a similar mechanism to protect themselves from B-0571 and B-1311 at the pupal stage is not known.

### Mortality of 3rd instar *Spodoptera frugiperda* vs *Helicoverpa armigera*

The overall mortality of 3rd instar *S. frugiperda* caterpillars infected with isolates B-0571 or B-1311 are higher than that of *H. armigera*. This suggests that either *H. armigera* has a higher resistance to fungal infection or it is not a target species of B-0571 and B-1311. A similar scenario was reported by Gutierrez et al. (2015) who showed *M. anisopliae* to be highly pathogenic towards oriental cockroach (*Blatta orientalis*)*,* but not to other cockroach species (e.g., German cockroach *Blattella germanica*). The authors reported at least 19 fatty acids on the outer layer surface of *B. orientalis* but not on *B. germanica*. The fatty acids were speculated to be a determinant of fungus virulence; however, the mechanism of how they facilitated *M. anisopliae* was not reported. Therefore, the lower susceptibility of *H. armigera* could be due to it lacking specific signals (e.g., fatty acids) on the cuticle such that the germination process is not triggered. Other factors that could contribute to the lower susceptibility of *H. armigera* may include the activation of antifungal mechanisms to increase protection against fungal infection, or production of secondary metabolites by B-0571 and B-1311 that better suited species within the *Spodoptera* genus, including *S. frugiperda*. This hypothesis could be tested by including endemic *Spodoptera* species e.g., *S. litura* and Australian *S. exigua* (a potentially cryptic species from African *S. exigua*; see Agarwal et al. 2022).

## Conclusion

Eleven fungal candidates from the CSIRO fungal collection were assessed for mortality induced in 3rd instar *S. frugiperda* caterpillars to guide selection of isolates of Australian EPF that showed promise for combating this newly arrived pest species. This could open up opportunities to develop alternative *S.* *frugiperda* pest management strategies, as well as to limit the usage and hence reduce negative environmental, biological, and ecological impacts of synthetic insecticides. The two most effective isolates, B-0571 and B-1311, were also effective against 6th instar caterpillars and adults of *S. frugiperda*, indicating that biological insecticides using these isolates may be possible to target both larval and adult life stages. Isolates B-0571 and B-1311 were both less effective against Australian endemic *H. armigera* than against *S. frugiperda.* Understanding how these fungal isolates impact other pest species could help to ascertain mechanisms of host specificity. Despite the promise of the EPF isolates identified through this study, other perspectives such as the production of secondary biomolecules that could be toxic to humans and beneficial insects must be taken into consideration. Further testing involving genomes, transcriptomic characterisation, and toxicity assessment in human cell lines/organoids should take precedence before any biopesticide development to minimise potential harmful, unwanted, and unintended exposures.

### Supplementary Information

Below is the link to the electronic supplementary material.

Supplementary file1 (FASTA 19 KB)

Supplementary file2 (FASTA 19 KB)

Supplementary file3 (DOCX 358 KB)

## Data Availability

Raw data generated during and/or analyzed in the present study and the statistically analysis code are available upon the request, the ITS sequences are available in GenBank (OM865403—OM865413).
