# Supplementary material for: Virulence of Beauveria sp. and Metarhizium sp. fungi towards fall armyworm (Spodoptera frugiperda)
Source: Arch Microbiol. 2023 Sep 7;205(10):328. doi: 10.1007/s00203-023-03669-8 (PMC10495518; doi:10.1007/s00203-023-03669-8)
Supplement: Supplementary file 3 — Supplementary file3 (DOCX 358 KB) [file 203_2023_3669_MOESM3_ESM.docx]

**9. Supplementary Data**

An in-house protocol of *S. frugiperda* semi-solid artificial diet was used for *S. frugiperda* caterpillar rearing (adapted from (Bowling 1967; Greene et al. 1976). Mixture A was prepared by combining 100g of navy bean flour, 25g of soy flour, and 600ml of filtered water and mixing with a stick blender. Mixture A was heated using a microwave to a boil (4 mins). In a separate measuring cup, mixture B was prepared by combining 18g of agar (GELITA, A-181017), 1.5g of sorbic acid (Sigma-Aldrich, S1626), and 500ml of filtered water and mixed with a spatula. Mixture B was heated using a microwave until boiling (8 mins). Mixture A and B were combined with a stick blender and allowed to cool down to 60℃ at room temperature. The remaining dry ingredients including 60g of wheat germ, 30g of brewers’ dry yeast, 3.3g of L-ascorbic acid (Sigma-Aldrich, A4403), 10g of Vanderzant vitamin mixture for insect (Sigma-Aldrich, V1007), 15g of casein (Sigma-Aldrich, C7078), and 3g of Methyl parahydroxybenzoate (Sigma-Aldrich, 79721) were added. Finally, 5ml of vegetable oil was added. All the ingredients were thoroughly combined with a stick blender. The diet was poured into 45-wells plates or 32-well plates while still warm, then allowed to set at room temperature for at least an hour. The diet was either used immediately or was stored at 4°C for a maximum of one week.

**10. Supplementary Figures**


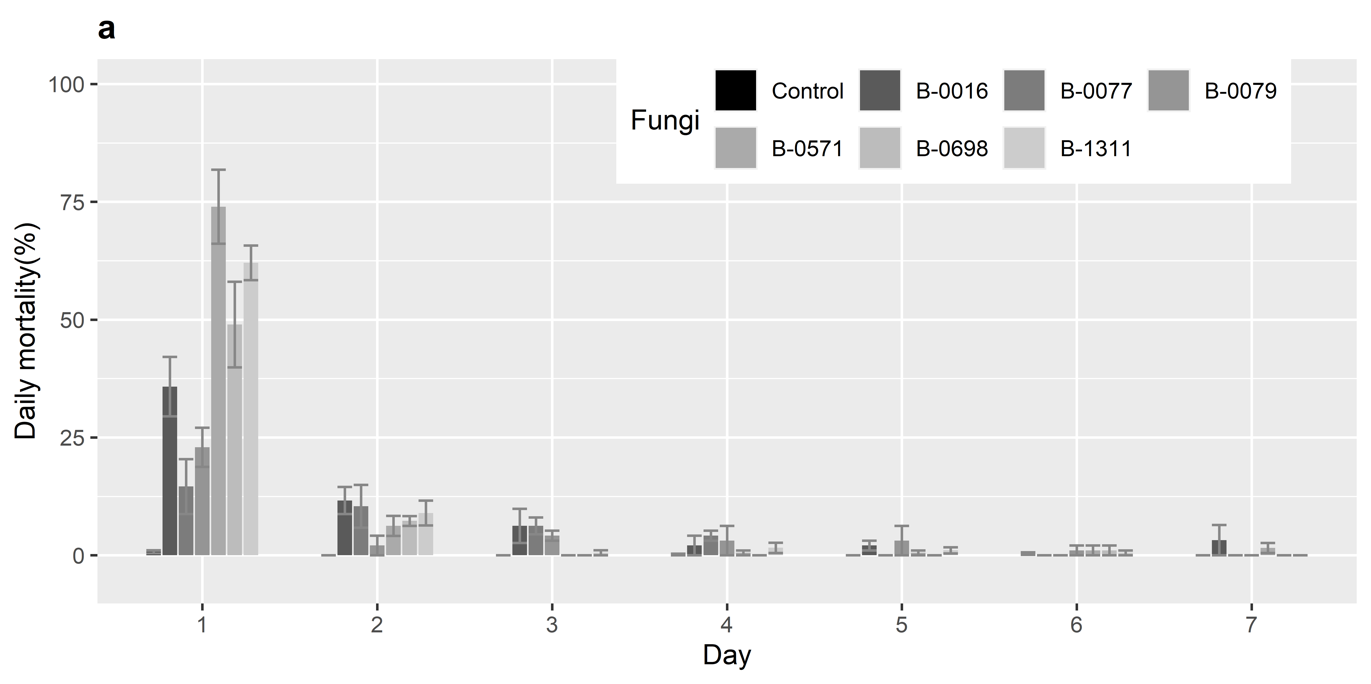

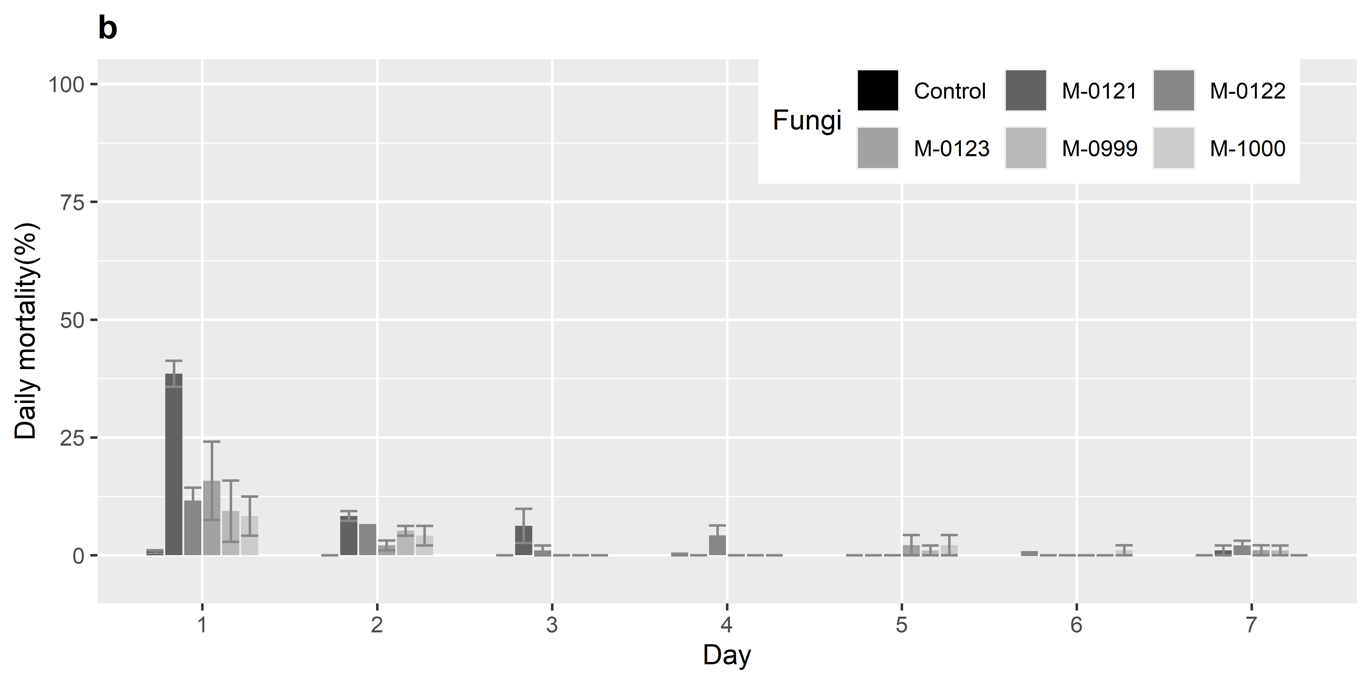


**Figure S1.** Daily mortality (%) of 3^rd^ instar *Spodoptera frugiperda* infected with a). *Beauveria* sp. and b). *Metarhizium* sp. for seven days after infection. The error bars indicate the standard deviation of the results and the various shades of grey correspond to the treatment (labelled in the right conner).


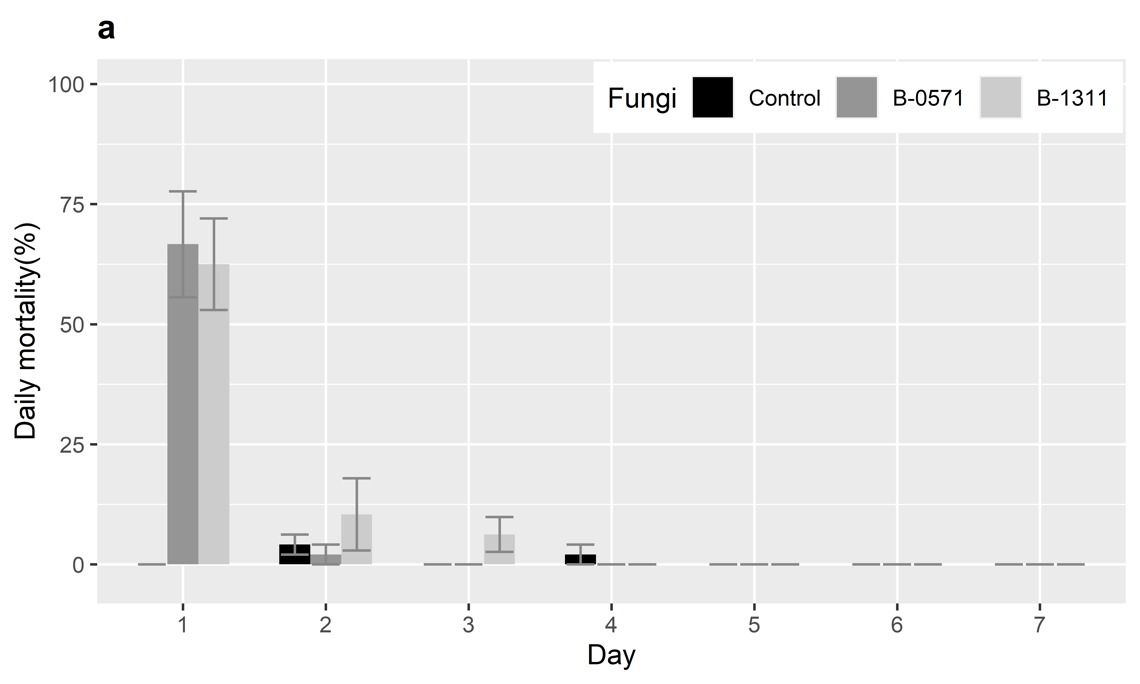


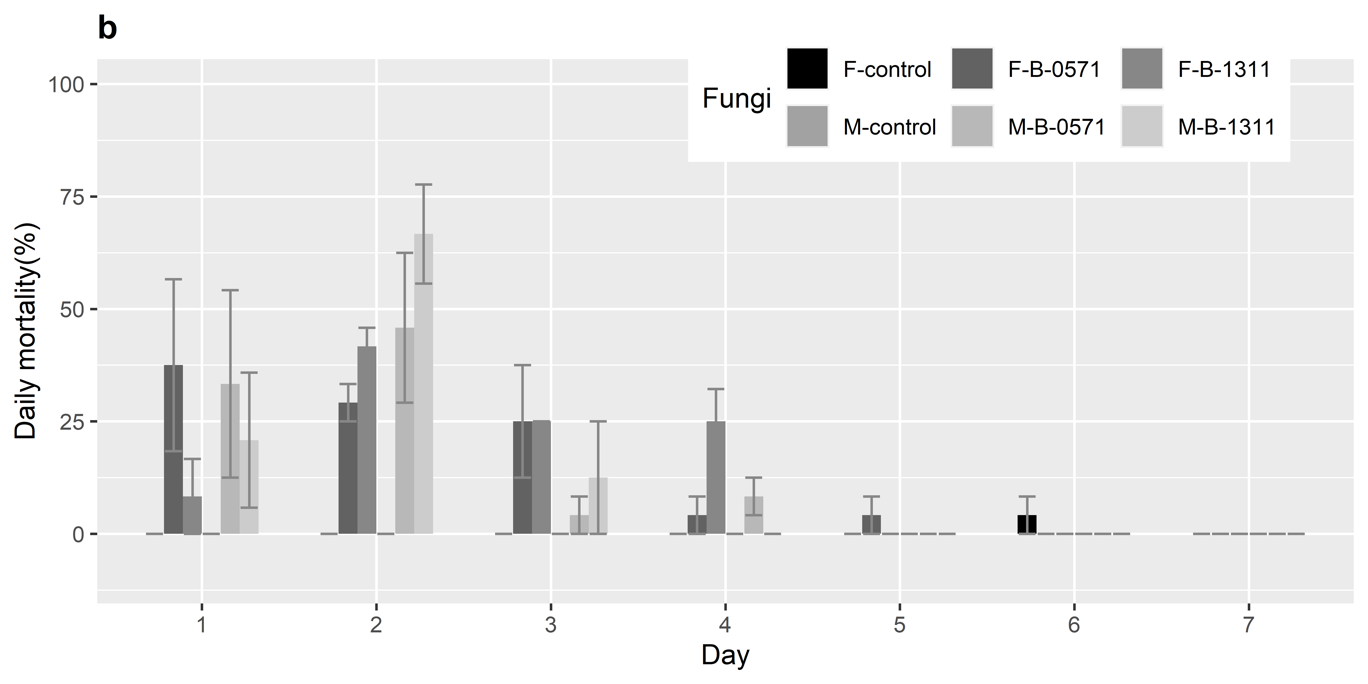


**Figure S2.** Daily mortality (%) of *Spodoptera frugiperda* at (a) 6^th^ instar caterpillars and (b) moth life stages for seven days following infection with *Beauveria* sp. (B-0571 or B-1311). The standard deviation is indicated as the error bars and the treatment and sex of study samples are specified by the shade of the bars (F = female and M = male).


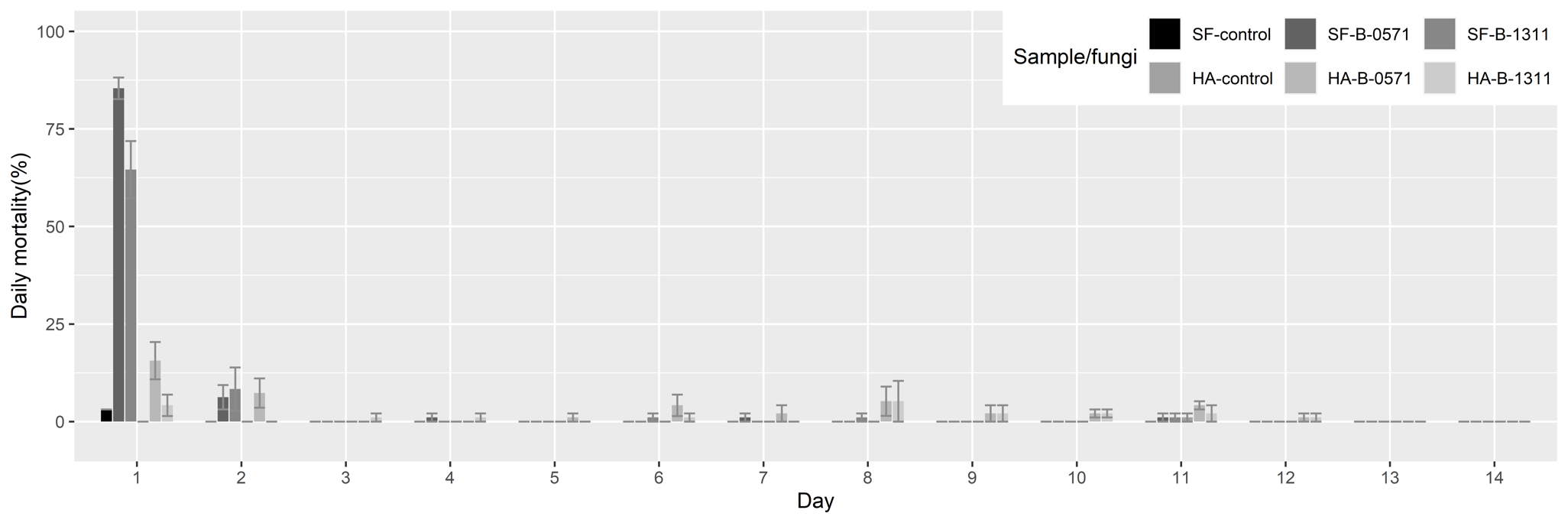


**Figure S3.** Daily mortality (%) of 3^rd^ instar *Spodoptera frugiperda* and *Helicoverpa armigera.* The error bars show the standard deviation and the shades of grey indicate the treatment including control or treated as well as species of the test samples (SF = *S. frugiperda* and HA = *H. armigera*).

**References**

Bowling CC (1967) Rearing of Two Lepidopterous Pests of Rice on a Common Artificial Diet. Ann Entomol Soc Am ANN ENTOMOL SOC AM 60:1215–1216. https://doi.org/10.1093/aesa/60.6.1215

Greene GL, Leppla NC, Dickerson WA (1976) Velvetbean Caterpillar: A Rearing Procedure and Artificial Medium123. J Econ Entomol 69:487–488. https://doi.org/10.1093/jee/69.4.487
